# The effects of perception of video image and online word of mouth on tourists’ travel intentions: Based on the behaviors of short video platform users

**DOI:** 10.3389/fpsyg.2022.984240

**Published:** 2022-09-08

**Authors:** Yang Zhou, Ligang Liu, Xiao Sun

**Affiliations:** ^1^Department of Business, Liaoning University, Shenyang, China; ^2^College of Culture and Tourism, Jiangsu University of Technology, Changzhou, China

**Keywords:** video images perception, online word of mouth, travel intentions, emotional evaluation, short video platform, cognitive image, affective image, gender

## Abstract

This research discusses the impact of the perception of video images and online word of mouth on tourists’ travel intentions. A survey of 390 users who watched travel videos on short-video platforms was conducted using structural equation modeling. The results are as follows. First, the perception of video images can significantly affect tourists’ intention to visit the destinations. Second, as a mediating variable, online word of mouth can enhance the positive effects of the perception of video images on tourists’ travel intentions. Third, gender had a positive moderating effect, which was particularly obvious in the relationship between the perception of video images and online word of mouth. This research provides a theoretical basis for the utility of tourism-related short videos, which can help stimulate tourists’ intention to visit promoted destinations.

## Introduction

The image of a tourist destination undoubtedly plays a significant role in its further development. As one of the crucial aspects of marketing a destination to attract consumers to visit, promoting a positive image plays a vital part in tourists’ post-tour evaluations and future intentions (Tu et al., 2017; Li et al., 2021; Jebbouri et al., 2022). The popularization of mobile internet and social media platforms has seen the gradual diversification of tourism promoters’ marketing methods. Tourists once obtained information on destinations through traditional media, such as newspapers, pictures, brochures, TV, and radio, but now they have turned to new outlets such as social media, online forums, and short-video platforms. In an era when “each person is a medium and uses media all the time,” tourism destinations and scenic spots mostly upload short videos to short-video social media platforms to heighten the possibility of potential tourists seeing their message, which can shape their image while stimulating tourists’ interest (Xin and Fei, 2020). In addition, destinations and tourist attractions actively encourage people to publish their own short travel videos, such as travel vlogs, a type of user-generated content which refers to users recording their travel notes and sharing their real travel life (Li et al., 2022). As one of the most important tourist sector marketing strategies, marketing through videos not only vividly presents the uniqueness of a given destination, but also undercuts the limitations of location and time to bring tourists an immersive experience (Chew and Jahari, 2014; Alamäki et al., 2022). Therefore, video-centered marketing strategies have become one of the main impetuses for the sustainable development of tourist attractions, as they are crucial to tourism enterprises’ efforts to increase their social and economic benefit.

The relationship between tourism destination image and tourists’ intention has become a hot topic in recent years (e.g., Yang et al., 2021, 2022a). Some scholars point out that image perception has become one of the most necessary factors in tourists’ decisions (Huete-Alcocer et al., 2019). In addition, there is growing scholarship on the role of short videos in forming the image of tourist destinations, including the functions of short videos in such image building (Jie, 2019), the psychological and behavioral intentions that short videos can trigger (Li et al., 2020; Liu and Yan, 2021; Xu et al., 2021), and the positive effects of the image cultivated by tourist videos on potential tourists’ intention to visit (Kim et al., 2018; Li et al., 2021). Short videos can meet consumers’ needs for personalized and video-based content sharing and expression, and the proportion of user-generated videos uploaded to short-video platforms has reached 42.8% (Zhang and Tan, 2022). A large number of studies, analyzing the topic from the perspective of the impact of short videos, have already confirmed that the image of a destination has a positive effect on tourists’ behavioral intentions (Kim et al., 2018; Li et al., 2020; Liu and Yan, 2021). However, few studies contain empirical surveys based on a large number of short-video platform users. Therefore, it is of great practical significance to analyze the impact of tourists’ perceptions of destinations generated from short videos on their travel intentions through a survey of short-video platform users.

The positive image perception of a tourist destination can attract tourists, trigger their positive evaluation and recommendations, and play an important role in forming tourists’ future travel intentions (Tu et al., 2017). According to tourism consumer praxeology, tourists cannot perceive the quality of a service or product before its consumption, thus they are prone to actively search for information before making decisions, and their decisions are affected by reputation (Leong et al., 2019). Massively shared, informative, and high-quality word of mouth reviews induce, to some extent, changes in consumer perceptions of the related destinations, thereby indirectly affecting consumers’ intention to visit them (Wang et al., 2019). Tourists’ intentions can be stimulated by not only the image of a destination but also their emotional evaluation generated by online word of mouth (Junaedi and Harjanto, 2020). It is worth noting that there is little research on the antecedent variables of emotional production in the current tourist emotional response literature (Cohen et al., 2014), and few scholars have included the image of tourist destinations and related online word of mouth in their analyses of tourist emotion formation (Cohen et al., 2014; Prayag et al., 2015; Qi et al., 2021). Although Prayag et al. (2015) notes the existence of this problem, the source of emotion and the emotional motivation behind tourists’ intentions have not been discussed in depth (Tu et al., 2017), and the mechanism behind emotional evaluation has not been clearly defined. Therefore, surveys of short-video platform users is of great theoretical and practical significance to exploring how the perception of video images affects online word of mouth and tourists’ intention to visit the destinations.

To answer this question, this study focuses on how the perception of video images can affect tourists’ intention to travel based on the theory of emotional evaluation. Online word of mouth evaluations caused by an emotional response to the perception of the destination image generated by the videos was adopted as a mediating variable to analyze its functional dynamic. The analysis was conducted using a structural equation modeling method with users who watched travel videos on short-video platforms as the objects of analysis.

This research contributes to the following three aspects of the field. First, empirical research is lacking on short-video platform users, whose number is consistently increasing. This research involves empirical research on users who watched travel videos on short-video platforms. It explores the impact of the image of tourist destinations generated by such videos on tourists’ travel intention, providing empirical evidence for the relationship between the two, and filling the gap in short-video user research. Second, the theory of emotional evaluation has been used to further reveal the mediating role of emotional evaluation, inspired by online word of mouth, between the image of tourist destinations generated by videos and tourists’ intention to visit them. Previous studies have rarely used online word of mouth as an intermediate variable to measure the dynamic between destination image and tourists’ intention, thus this study expands the research on online word of mouth and related fields. Lastly, this research can help in the formulation of relevant tourism management policies and the effective management of the image of tourist destinations. The research results can also be of considerable significance in the formulation of precision-targeted short video marketing strategies, and of the high-quality development of tourist destinations and attractions.

## Literature review and hypotheses development

### The theory of emotional evaluation

Emotional evaluation theory, which originated in the field of psychology, is often used to explain the adaptive response of individual emotions to external environmental stimuli (Lazarus, 1991; Siemer et al., 2007). Specifically, it argues that emotions usually derive from personal opinions and subjective evaluations, which are in turn formed by individuals’ cognition of the surrounding environment and events, and by their resultant motivations (Huang et al., 2020). The theory has been applied to the field of consumer behavior and has been markedly promoted in the field of marketing through identifying the antecedent and outcome variables of consumer emotions (Watson and Spence, 2007). Some scholars have applied this theory to network public opinion governance (Liu et al., 2021), customer satisfaction and brand loyalty (Susanti et al., 2020), and video images (Zaretsky et al., 2010) among others.

Regarding tourism, Hosany (2012) introduced the theory of emotional evaluation to tourist behavior research, and its prevalence in the tourism field has been increasing ever since. For example, Liang (2021) analyzed the development of rural tourism with local customs in terms of consumer satisfaction. Munanura et al. (2021) discussed the role of attitudes, emotional solidarity, and subjective wellbeing on tourism development. Fang and Tang (2020) found that the “cognition-emotion-intention-behavior” pathway constructed by emotional evaluation theory can organically combine these four elements. In other words, it can better demonstrate that tourists often generate a series of cognitive and emotional evaluation behaviors when perceiving the cognitive and affective images of a tourist destination, which not only induces their intention to visit but also certain travel behaviors. This study contends that emotional evaluation theory can productively explain the mechanism behind tourists’ travel intentions in the online word of mouth context, stimulated by their perception of the image of a tourist destination generated by videos. The recommendations and sharing behaviors inherent to online word of mouth can be regarded as an evaluation behavior aimed at sharing knowledge, with information about destinations as the carrier (Qi et al., 2021). The interactive information-sharing behavior of uploading videos of tourist destinations to short-video platforms, generating positive or negative word of mouth, is not only related to users’ experience and emotions (Echeverri and Åkesson, 2018), but also to whether tourists’ demands have been satisfied. Travel intentions are closely related to these factors (Tsaur and Teng, 2017). Therefore, emotional evaluation theory provides a suitable theoretical framework for exploring the influences of destination perception from video images on tourists’ intention to visit.

### Destination perception of video images and online word of mouth

The perception of a tourist destination’s image has been regarded as the sum of individuals’ trust in the information they received about it, their opinions about it, and their impressions and expectations of it. A positive image overcomes the limitations of space and time, and fully evokes the attractiveness of a tourist destination, thus establishing a feeling of immersion in that image in potential tourists (Li et al., 2019, 2021). The cognitive and affective dimensions have been used primarily in assessing the status of a destination’s image in the existing literature (Stylidis, 2016; Stylidis et al., 2017a), and it has been considered that these dimensions have strong explanatory power (Stylidis et al., 2017b; Wei, 2020; Li et al., 2021). “Cognitive image” denotes the impression potential tourists have of the various attributes of a destination, including its infrastructure, natural scenery, history, and culture (Stylidis et al., 2017b; Yang et al., 2022b). “Affective image” refers to tourists’ subjective emotional response to their perception of a destination, which is often expressed by adjectives such as “interesting” or “spectacular” (Marine-Roig and Ferrer-Rosell, 2018; Molinillo et al., 2018). Thus, this study also divides the image of destinations generated by short videos into cognitive and affective images.

Generating and disseminating online word of mouth is primarily based on the internet as a media platform, and the resulting decision-making behaviors of consumers are derived from the exchange of information between them on this basis (Pourfakhimi et al., 2020). This context has the characteristics of immediacy, anonymity, the durable retention of information, the exertion of powerful influence, and freedom from the constraints of space and time, among others (Ma et al., 2015). Regarding research on online word of mouth, some scholars have analyzed its content in terms of quantity relative to specific destinations, the bias it displays, and the evaluations it receives (Cui et al., 2012; Liang et al., 2021), while others consider quality and quantity as its most important dimensions (Wei, 2020; Yan, 2020). The volume of online word of mouth content, also known as the number of online reviews, represents the degree of attention the evaluated item has received, and also implies the important factors that consumers consider in their decision-making (Park et al., 2007; Cui et al., 2012; Liang et al., 2021). Its quality is usually judged based on the quality of the evidence used and the reliability of its source (Huang and Lao, 2013), and it can exert a direct impact on information recipients (Filieri et al., 2015). The composition of online word of mouth has multi-dimensional attributes, depending on the research objective; however, the most basic attributes are often seen as its quality and quantity (Yan, 2020). Therefore, this study takes quality and quantity, the relatively stable characteristics of online word of mouth, as its two primary dimensions.

Based on an analysis of the relationship between a destination’s image perception and the associated online word of mouth, it can be concluded that the latter, which is formed by the sharing of tourists’ experiences on social media platforms, has become a new path for the dissemination of the image of tourist destinations (Ma et al., 2018; Zhao et al., 2018), and the relationship between the two is complementary. A positive image helps a destination build positive online word of mouth, while negative online word of mouth reduces tourists’ expectations of a specific destination (Yang, 2018). Emotional evaluation theory contends that people produce a series of cognitive evaluations based on the stimulation produced by a certain situation, and the content of relevant evaluations can cause a variety of reactions (Moors, 2020). Hence, the perception of the image of a tourist destination generated by videos can play a positive role in the evaluations generated in online word of mouth. Specifically, the more positive the perception of the image of a tourist destination generated by videos, the more likely online word of mouth will have a positive effect, and that the quantity of positive word of mouth will increase (Jing et al., 2015). Hence, the image of a destination generated by videos is clearly related to the quality of its online word of mouth (Kim et al., 2017). Meanwhile, Yang (2018) has also pointed out that both cognitive and affective images of a destination have a positive impact on its word of mouth evaluations. Based on the above analysis, the following hypotheses are proposed:

H1a: The cognitive perception of video images has a positive effect on the quality of a destination’s online word of mouth.

H1b: The cognitive perception of video images has a positive effect on the quantity of a destination’s online word of mouth.

H1c: The affective perception of video images has a positive effect on the quality of a destination’s online word of mouth.

H1d: The affective perception of video images has a positive effect on the quantity of a destination’s online word of mouth.

### Destination perception of video images and tourists’ travel intentions

Travel intentions are usually regarded as a type of tendency to travel that has not yet been actualized; that is, the attitude and possibility of a tourist to go to a certain destination in the future (Liang, 2019). It is considered the basis of and premise for a tourist participating in tourism activities (Bin, 2019), and also as a positive response to the image of a tourist destination (Wang, 2021). Emotional evaluation theory posits that the process of cognitive evaluation, based on an emotionally charged event, can exert direct effects on individual attitudes and behaviors (Lin et al., 2019; Moors, 2020). This indicates that the image of a certain destination is very important to a tourist because their perception of it can serve as a direct influencing factor on their travel intentions, recommendation behaviors, and intention to revisit (Prayag et al., 2015; Junaedi and Harjanto, 2020; Yang et al., 2020b). Tourists perceive an image of a tourist destination generated by videos, and their decision-making process and formation of intended behavior are further influenced by that perception (Cheng et al., 2020). The relationship between the image of tourist destinations generated by videos and tourists’ travel intentions has attracted much scholarly attention, with relatively consistent conclusion. For example, Adeloye et al. (2021) found that vlogs can significantly impact tourist travel intentions. Silaban et al. (2022) argued that watching travel vlogs on YouTube can significantly affect consumers’ travel intentions. In terms of the ways in which the image of a tourist destination generated by videos can significantly and positively motivate tourists to visit (Fu et al., 2016; Li et al., 2021). Moreover, the cognitive image of a destination can help predict tourists’ travel intentions (Stylidis et al., 2017a; de la Hoz-Correa and Muñoz-Leiva, 2019; Woosnam et al., 2020), while its affective image can play a positive role in their decision-making process (Han et al., 2019; Afshardoost and Eshaghi, 2020; Akgün et al., 2020). Based on these findings, the following hypotheses are proposed:

H2a: The cognitive perception of video images has a positive effect on tourists’ travel intentions.

H2b: The affective perception of video images has a positive effect on tourists’ travel intentions.

### Online word of mouth and tourists’ travel intentions

With the rapid development of social media platforms, an increasing number of potential tourists have chosen to disseminate travel-related information online to make wise decisions (Zhu et al., 2021). Emotional evaluation theory argues that the various emotional responses caused by cognitive evaluations can predict to some extent individual behaviors and attitudes (Lin et al., 2019; Moors, 2020). Thus, tourists are often affected by the attitudes of other tourists when assimilating relevant information on tourist destinations, and online word of mouth about a destination plays a critical role in consumers’ decision-making process in relation to their travel intentions (Qi et al., 2021). Specifically, the quantity, professionalism, and reliability of online word of mouth directly impact tourists’ decision-making, and ultimately affect their travel intentions (Wei, 2020; Thaothampitak and Wongsuwatt, 2022). Moreover, positive word of mouth enhances their travel intentions, while negative word of mouth directly moderates them (Zhu et al., 2021). Furthermore, the quality of online word of mouth can not only immediately affect tourists’ trust in the information conveyed (Wei, 2020) but also strengthen their travel intentions (Ting et al., 2018; Andriani et al., 2019). Accordingly, it is assumed that online word of mouth evaluation, triggered by the emotional event of the perception of the image of a tourist destination generated by videos, can help predict and affect tourists’ travel intentions. Therefore, the following hypotheses are proposed:

H3a: The quality of online word of mouth has a positive effect on tourists’ travel intentions.

H3b: The quantity of online word of mouth has a positive effect on tourists’ travel intentions.

### The mediating role of online word of mouth between the destination perception of video images and tourists’ travel intentions

A tourist tends to give a positive evaluation of a tourist destination if their experience of it during their trip is consistent with their expectations (Tu et al., 2017). Their perception of the destination’s image helps predict whether a tourist will recommend it and their intentions to revisit (Hosany et al., 2017; Chaulagain et al., 2019). According to the theory of emotional evaluation, an individual’s emotional evaluation stimulated by a certain event can not only induce specific behaviors but also indirectly affect others’ behaviors by influencing individual attitudes (Moors, 2020). Thus, the emotional evaluation responses resulting from the perception of the image of a tourist destination generated by videos, indirectly induce certain behaviors from tourists. Studies have shown that when online word of mouth interacts with the image of a tourist destination, it alters tourists’ intentions to visit it and plays a mediating role between the two (Junaedi and Harjanto, 2020; Wei, 2020). When the destination is positively evaluated through online word of mouth, the relationship between the perception of the image of a destination and consumers’ travel intentions becomes more significant (Farrukh et al., 2020). For example, a tourist’s perception of the image of a destination may change after assimilating online word of mouth evaluations about the destination (Litvin et al., 2018). This is because the tourist’s perception, travel intentions, and evaluation are modified when the destination develops a negative image through online word of mouth (Zhu et al., 2021). Meanwhile, the tourist’s intentions to choose to visit this destination or scenic spot is also weakened (Hou et al., 2020). In contrast, positive online word of mouth on social media platforms inspires more tourists to travel to that destination (Liu et al., 2019). Therefore, the following hypotheses are proposed:

H4a: Online word of mouth mediates the relationship between the cognitive perception of video images and tourists’ travel intentions.

H4b: Online word of mouth mediates the relationship between the affective perception of video images and tourists’ travel intentions.

### The moderating effect of gender

Continuous improvement in the verification of mediating effects has helped shift scholars’ attention to the moderating effect of variables. Especially in behavioral research, gender differences are often involved in all aspects of tourists’ intention to travel and decision-making (Pan et al., 2020; Carballo et al., 2021), and play a significant role in the marketing strategies of tourist destinations (Pan et al., 2021). Travel activities are often considered a social phenomenon, constructed on the basis of tourists’ genders. There is an inextricable relationship between tourism-related perceptions and gender in the travel process, and social psychology studies have indicated that there are obvious differences between females and males in terms of their emotional experience and behavioral decision-making relating to tourism (Jia et al., 2018; Ahn et al., 2020). Huang and Veen (2019) found noticeable differences between men and women regarding the impact of a tourist destination’s image on travel intentions. Men usually attach more importance to the available services at a destination than women, while women place much more priority on the natural environment.

There are also certain disparities between men and women regarding how they evaluate information on the internet (Abubakar et al., 2016). Women tend to value the quality and usefulness of information more than men when assimilating online word of mouth evaluations (Torres et al., 2018), and it has been argued that browsing online word of mouth reviews helps women make faster decisions (Dedeoglu, 2019). Therefore, even in the same environment, individual differences result in different emotional responses to environmental stimuli (Lazarus, 1991; Huang and Korfiatis, 2015). Emotional evaluation theory contends that the process of inducing individual emotions through external environmental stimuli can be divided into the inducement of an initial evaluation and then of a re-evaluation (Lazarus, 1991; Huang and Korfiatis, 2015). In the initial evaluation process, an individual is subject to the influences of external stimuli, which involves the evaluation of tourist destinations and their relevance to the individual, while the re-evaluation process can be seen as the reprocessing of online word of mouth based on the initial evaluation. The initial evaluation is altered by adjusting the individual’s stimulus response to the external environment in this process, which can function to regulate the intensity of their emotions (Lazarus, 1991; Huang and Korfiatis, 2015). In this study, the perception of video images, online word of mouth, and tourists’ travel intention all represent to various extents the individual’s response to external stimuli. Therefore, the following hypotheses are proposed to explore the role of gender in the perception of video images, online word of mouth, and travel intentions:

H5: Gender has a moderating effect on the strength of the relationship between the perception of video images and online word of mouth.

H6: Gender has a moderating effect on the strength of the relationship between online word of mouth and tourists’ travel intentions.

H7: Gender has a moderating effect on the strength of the relationship between the perception of video images and tourists’ travel intentions.

**FIGURE 1:**
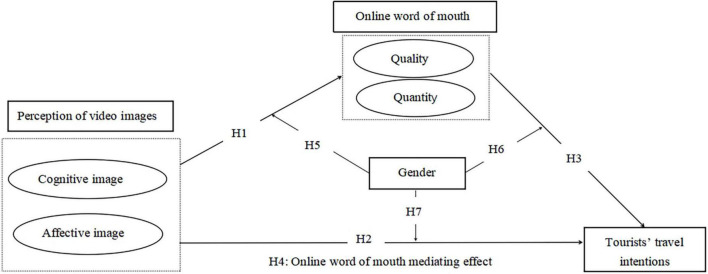
The research model.

The research model in this study is shown in Figure 1.

## Methodology

### Sample

This study primarily explores the influence of the perception of video images on tourists’ travel intentions. The survey respondents were users who had watched tourism-related videos on short-video platforms through devices, including mobile phones and computers. A random sampling method was followed and online questionnaires were developed through Question Star. In the period from October 10th to the 31st, 2021, 476 questionnaires were distributed online through platforms such as WeChat, Weibo, and Douyin (the Chinese version of TikTok), among others. After excluding the questionnaires in which respondents gave the same answers to all questions or had excessively short response times, 390 valid questionnaires were finally obtained, with an effective rate of 81.9%. The results are shown in Table 1. Based on the respondents’ demographic information, 46.2% were female and 53.8% were male. Regarding age, the proportions of respondents under 20 years old, 20–30 years, 31–40 years, 41–50 years, and over 50 years were 13.8, 42.8, 23.1, 14.4, and 5.9%, respectively. The proportions of respondents under 20 years, 41–50 years, and over 50 were smaller than those of other age groups, likely owing to either these groups’ lower level of familiarity with the internet, or the amount of time they spend on short-video platforms. Regarding respondents’ educational background, those with a junior college degree and lower education, a bachelor’s degree, a master’s degree, or a doctor’s degree were 16.6, 62.1, 12.8, and 8.5%, respectively, indicating that the research subjects were able to sufficiently understand the questionnaire content, thereby ensuring the reliability of the survey data. Regarding occupation and monthly income, students accounted for 40.5%, corporate employees 16.4%, and freelancers 9.5% of the sample. Respondents whose monthly income was below 2,000 yuan accounted for 34.4% of the sample, those with more than 5,000 yuan 30.5%, and those with 4,001–5,000 yuan 12.3%. Considering that the respondents in the sample were mostly young or middle-aged individuals, the data distribution of the sample was basically reasonable. When asked about the platforms or sites where they watch travel videos, 71.5% stated they use professional travel websites, 66.2% use self-media platforms, and 54.1% use social networking platforms. Regarding the video channels that respondents trust, professional travel websites accounted for 70%, self-media 54.4%, and social networking platforms 48.2%. Regarding the channels they use for video sharing, 60% of them use social media platforms, 58.2% use self-media, and 53.3% use professional travel websites. Therefore, it can be concluded that the respondents engage in a high level of activity on professional travel websites, self-media platforms, and social networking platforms.

**TABLE 1:** The composition and distribution of the sample (*N* = 390).

Item	Category	Frequency	Rate%	Item	Category	Frequency	Rate%
Gender	F	180	46.2	Source channel	Professional travel websites	279	71.5
	M	210	53.8		Portal sites	169	43.3
Age	Under 20	54	13.8		Self-media	258	66.2
	20–30	167	42.8		Social media platforms	211	54.1
	31–40	90	23.1		Online community sites	111	28.5
	41–50	56	14.4		Others	71	18.2
	Over 50	23	5.9	Trusted channel	Professional travel websites	273	70
Education	Less than high school/technical school	31	7.9		Portal sites	146	37.4
	Junior college	34	8.7		Self-media	212	54.4
	Bachelor’s degree	242	62.1		Social media platforms	188	48.2
	Master’s degree	50	12.8		Online community sites	104	26.7
	Doctor’s degree	33	8.5		Others	75	19.2
Occupation	Student	158	40.5	Sharing channel	Professional travel websites	208	53.3
	Civil servant/public institutions	37	9.5		Portal sites	124	31.8
	Corporate employee	64	16.4		Self-media	227	58.2
	Lawyer/teacher/healthcare professional	64	16.4		Social media platforms	234	60
	Freelancer	37	9.5		Online community sites	82	21
	Others	30	7.7		Others	69	17.7
Monthly income (yuan)	Under 2000	134	34.4				
	2001–3000	43	11				
	3001–4000	46	11.8				
	4001–5000	48	12.3				
	Over 5000	119	30.5				

### Measurement of variables

Based on the research model above, a measurement scale was designed with reference to highly cited domestic and international research and using mature scales. The content of the questionnaires was adjusted based on the characteristics of this study. The first part of the questionnaire relates to the perception of video images, with a total of 16 items in the two dimensions of cognitive and affective image. To measure the perception of video images, this study uses relevant items from the measurement scales in Stylidis et al. (2017b) and Li et al. (2021), which have been widely applied in empirical research on the perception of the cognitive and affective image of tourist destinations and on consumer travel intentions. These scales were verified as having good utility. The second part of the questionnaire relates to the effects of online word of mouth, with 13 items in the two dimensions of quality and quantity. This study employs the scales from Park et al. (2007), Huang and Lao (2013), and Wei (2020), which focus on exploring the relationship between destination image, online word of mouth quantity and quality, and tourists’ travel intention. These scales have also been evaluated as having good reliability and validity. The third part relates to tourists’ travel intentions. Drawing on the scales employed in the studies of Fu et al. (2016) and Tu et al. (2017), which examine the process through which the image of a tourist destination influences tourists’ intention to visit, this study uses four items to measure travel intentions. The fourth part comprises the study’s control variables. According to existing research, demographic variables have a significant impact on tourists’ travel intentions (Almeida and Moreno, 2018). Therefore, this study takes gender, age, education, occupation, monthly income, channels for viewing videos, trusted channels, and channels used for sharing as the control variables, from which the channels used for viewing and sharing videos, and those in which most trust is placed, are given in multiple choice format to facilitate respondents in making their choices.

To ensure the reliability and validity of each item in the questionnaire, three experts and five tourism enterprise managers were invited to conduct the first round of pre-testing before its official distribution. Some items in the questionnaire were modified based on their feedback. After 20 undergraduate and post-graduate tourism management and marketing management students conducted the second-round of pre-testing, the final version of the questionnaire was delivered. All latent variables in this study were measured on a 5-point Likert scale (1 = strongly disagree, 2 = disagree, 3 = uncertain, 4 = agree, and 5 = strongly agree).

## Results

### Confirmatory factor analysis

#### Reliability and validity test

Data analysis and processing was performed mainly through SPSS 26.0 and AMOS 26.0 software. The reliability of the questionnaire was initially analyzed using Cronbach’s alpha in SPSS 26.0, and the dimensions of the perception of video images, online word of mouth, and tourists’ travel intentions were constructed through exploratory factor analysis. Next, a confirmatory factor test was conducted through AMOS 26.0 on these three latent variables to further verify the stability and consistency of the scale. The test results are shown in Table 2. The Cronbach’s alphas of the cognitive and affective images of tourist destinations generated by videos were 0.926 and 0.868, respectively, and those of the quality and quantity of online word of mouth were 0.866 and 0.901. Finally, the Cronbach’s alpha of the effect of tourists’ travel intentions was 0.835. All the values were higher than 0.7, thereby meeting the standard acceptance criteria and demonstrating a high level of scale reliability.

**TABLE 2:** Confirmatory factor analysis.

Observed variable	Factor loading	*T*-value	CR	AVE	References
**Cognitive image** (Cronbach’s alpha **=** 0.926)					Stylidis et al., 2017b; Li et al., 2021
The destination in the videos has beautiful natural scenery.	0.707		0.925	0.508	
The destination in the videos has a unique historical and cultural landscape.	0.729	17.146			
The residents of the destination in the videos have unique lifestyles and customs.	0.723	18.619			
The destination in the videos has a pleasant climate.	0.806	15.196			
The destination in the videos has good public order.	0.808	15.154			
The destination in the videos has diverse foods.	0.731	13.783			
The destination in the videos has a fun nightlife.	0.721	13.581			
The destination in the videos has a clean environment.	0.704	13.283			
The businesses at the destination in the videos have standard transaction procedures and their prices are reasonable.	0.577	11.083			
There are standardized services offered by the businesses at the destination in the videos.	0.715	13.493			
The destination in the videos has convenient transportation.	0.699	13.106			
The destination in the videos has comprehensive and user-friendly infrastructure.	0.606	11.530			
**Affective image** (Cronbach’s alpha = 0.868)					
The destination in the videos would be relaxing.	0.827		0.875	0.637	
The destination in the videos would be exciting.	0.850	19.437			
I think being at the destination in the videos would make me happy.	0.818	18.517			
I think being at the destination in the videos would be an emotional experience for me.	0.685	14.666			
**Quality** (Cronbach’s alpha = 0.866)					Park et al., 2007; Huang and Lao, 2013; Wei, 2020
The online evaluations of the destination in the videos are consistent.	0.767		0.867	0.520	
I attach great importance to positive online word of mouth about the destination in the videos.	0.653	13.025			
The online evaluations of the destination in the videos are positive overall.	0.718	14.351			
The online word of mouth generated by the videos of the destination encourage me to go there.	0.750	15.149			
The online word of mouth about the destination generated by the videos is easy to understand.	0.689	13.762			
The online evaluations of the tourist destination generated by the videos show the real feelings of the tourists who have visited.	0.743	14.908			
**Quantity** (Cronbach’s alpha = 0.901)					
The online evaluations which were generated by videos of the destination, and which garnered a large number of user comments and replies, attract my attention more.	0.686		0.901	0.567	
A large amount of online word of mouth information on the destination in the videos that can be found on various online platforms.	0.619	13.777			
The online word of mouth evaluations of the destination in the videos on multiple platforms.	0.831	15.177			
The online word of mouth evaluations of the destination generated by videos being forwarded on multiple online platforms.	0.785	14.340			
The quantity of online reviews makes it easier for me to develop trust in the accuracy of their perspectives.	0.814	14.888			
I am more likely to read well-written online reviews.	0.798	14.553			
Images, videos and other forms of review are more attractive to me than simple text.	0.716	13.011			
**Tourists’ travel intentions** (Cronbach’s alpha = 0.835)					Fu et al., 2016; Tu et al., 2017
There is a good chance I will visit the destination after watching the videos about it.	0.785		0.829	0.552	
The destination is worth my time and money after watching the videos about it.	0.834	16.530			
I will sincerely recommend the destination in the videos to others after watching them.	0.714	13.999			
I will pass on a positive message about the destination to others after watching the videos.	0.622	12.155			

The validity test consists of two parts: convergent validity and discriminant validity. The test was conducted based on KMO, AVE, and other indicators, and shows that the KMO values were 0.935, 0.920, and 0.777, and the significance levels based on Bartlett’s test of sphericity were all 0.000, indicating that each variable is suitable for factor analysis. Meanwhile, the factor loading of each observed variable ranged from 0.577 to 0.850, each of which is larger than the recommended value of 0.5. The range of CR values was from 0.829 to 0.925, much higher than the standard value of 0.6. The AVE values of each dimension of the latent variables were 0.508, 0.637, 0.520, 0.567, and 0.552 respectively, all higher than the recommended value of 0.5. Therefore, the scale has good convergent validity. The validity test further showed that the scale has great accuracy and stability. In addition, as shown in Table 3, only the correlation coefficient of the quality and quantity of online word of mouth was slightly smaller than the AVE square root; however, this does not reduce the model’s good discriminant validity (Sim, 2013). More in-depth research can be conducted on this basis.

**TABLE 3:** Correlation coefficient and results of discriminant validity test.

Variable	Cognitive image	Affective image	Quality	Quantity	Travel intentions
Cognitive image	**0.712**				
Affective image	0.660	**0.798**			
Quality	0.551	0.680	**0.721**		
Quantity	0.411	0.519	0.730	**0.752**	
Travel intentions	0.423	0.521	0.643	0.666	**0.742**

The numbers in bold which proceed diagonally across the table are the AVE square roots, and the other numbers are the correlation coefficients between variables.

#### Goodness of fit test

Based on the results of the reliability and validity test, the parameters of the constructed model were examined using the maximum likelihood method in AMOS 26.0, selecting the following indices to test goodness of fit (Sim, 2013): *x*^2^/*df*,*CFI*,*NFI*,*GFI*,*AGFI*,*IFI*,*RMSEA*. The revised model was finally determined after modification. As shown in Table 4, the index values of the revised model have been improved to a certain extent, and have all reached the level of standard values compared with the adaptive standard values, which indicates that the model adaptation has reached the appropriate level.

**TABLE 4:** Goodness of fit test.

Index	*x^2^*/*df*	*CFI*	*NFI*	*GFI*	*AGFI*	*IFI*	*RMSEA*
Initial model index value	3.784	0.839	0.794	0.778	0.744	0.840	0.085
Revised model index value	2.378	0.924	0.876	0.857	0.827	0.924	0.060
Adaptive standard value	<5	>0.850	>0.850	>0.800	>0.800	>0.850	<0.080

### Path analysis and hypotheses testing

#### Path analysis

Before hypotheses testing, Harman’s single factor test was used to analyze the common method bias problem and an exploratory factor analysis was conducted on all items. The unrotated results show that all items are automatically aggregated into six factors with eigenvalues greater than 1. Their cumulative variance contribution is 66.515. The value of the first eigenvalue is 13.94, with a variance contribution rate of 42.244, which does not account for half of the total explanatory variables, and the values of the variance inflation coefficient (VIF) were all less than two. Thus, it can be seen that common method bias and the presence of collinearity do not exert serious influence in this study.

Based on the confirmatory factor analysis, the maximum likelihood method in AMOS 26.0 was used to analyze whether the proposed hypotheses are supported by the parametric test structure. Based on the results, shown in Table 5, the normalized path coefficients of H1a, H1b, H1c, and H1d were 0.561 (*t* = 11.533, *p* < 0.001), 0.439 (*t* = 7.650, *p* < 0.001), 0.180 (*t* = 3.078, *p* < 0.001), 0.180 (*t* = 3.078, *p* < 0.001), and 0.121 (*t* = 2.106, *p* < 0.05), respectively, indicating that the two dimensions of the perception of video images have a significant positive impact on the two dimensions of online word of mouth. Therefore, H1a, H1b, H1c, and H1d were proven. The H2a hypothesis showed a standardized path coefficient of 0.119 (*t* = 1.976, *p* < 0.05), indicating that the cognitive perception of video images has a significant positive influence on tourists’ travel intentions. However, the standardized path coefficient of H2b was 0.048 (*t* = 0.959, *p* > 0.1), thus the affective perception of video images does not significantly affect tourists’ intentions to visit. In addition, the standardized path coefficients of H3a and H3b were 0.246 (*t* = 4.794, *p* < 0.001) and 0.431 (*t* = 9.915, *p* < 0.001), suggesting that both the quality and quantity of online word of mouth can exert a notable positive effect on tourists’ travel intentions. The path coefficient results of the above analyses have statistically significant impact, which preliminarily supports the mediating effect of online word of mouth in the relationship between the perception of video images and tourists’ travel intentions.

**TABLE 5:** Hypotheses testing results.

Hypothetical path	Standardized path coefficient	S.E.	C.R.	*p*	Results
H1a: Cognitive image → quality of online word of mouth	0.561	0.053	11.533	***	yes
H1b: Cognitive image → quantity of online word of mouth	0.439	0.064	7.650	***	yes
H1c: Affective image → quality of online word of mouth	0.180	0.048	3.708	***	yes
H1d: Affective image → quantity of online word of mouth	0.121	0.057	2.106	0.035*	yes
H2a: Cognitive image → tourists’ travel intentions	0.119	0.066	1.976	0.048*	yes
H2b: Affective image → tourists’ travel intentions	0.048	0.05	0.959	0.337	no
H3a: Quality of online word of mouth → tourists’ travel intentions	0.246	0.052	4.794	***	yes
H3b: Quantity of online word of mouth → tourists’ travel intentions	0.431	0.043	9.915	***	yes

*p < 0.05, ***p < 0.001.

#### Mediating effect analysis

The bootstrap method in AMOS 26.0 was used to test whether there is a mediating effect for online word of mouth between the cognitive and affective perceptions of video images and tourists’ travel intentions. To begin with, the 390 valid samples in this study were adopted as the bootstrap population, and 5000 times of random selection were executed. Whether the interval included 0 through the 95% confidence interval of the mediating effect was then evaluated, and if not, the significance of the mediating effect could be indicated. Through the bootstrap (5000) test, online word of mouth was found to have a significant indirect effect on the relationship between tourists’ exposure to cognitive and affective video images of destinations and their intention to visit. As 95% of the confidence interval did not contain 0, the mediating effect was proven. Therefore, H4a and H4b were validated. The results are shown in Table 6.

**TABLE 6:** Bootstrap test results of the significance of the mediating effect.

Mediating effect path	Significance (two-tailed test)	95% of confidence interval	
		Lower limit	Upper limit
H4a: Cognitive image → online word of mouth → tourists’ travel intentions	0.000	0.262	0.423
H4b: Affective image → online word of mouth → tourists’ travel intentions	0.000	0.317	0.494

#### Moderating effect analysis

To investigate the moderating effect of gender on the variables (H5, H6, H7), this study employed a structural equation modeling-based multi-group analysis, which shows whether there are differences in the path coefficients between different gender groups. Furthermore, to present the comparative results more intuitively, it the difference ratio was defined as the percentage value of the difference between male and female groups and the path coefficient of the total sample. The results are shown in Table 7. Based on the hypothesis test results of H5, H6, and H7, it can be concluded that there is significant difference between the female and male groups in terms of the relationship between the perception of video images and online word of mouth. The path coefficient of the female group (β = 0.713) was higher than that of the male group (β = 0.598), thus H5 proves to be valid. Furthermore, gender also exerts a significant positive moderating effect on the relationship between online word of mouth and tourists’ travel intentions. The difference ratio was 1.1%, and the path coefficient of the male group (β = 0.622) is slightly lower than that of the female group (β = 0.629). Therefore, H6 proves to be valid. However, the moderating effect of gender on the relationship between the image of tourist destinations generated by viewing videos and tourists’ travel intentions was not very notable; thus, it was proven to have no moderating effect and H7 is not valid. To conclude, the moderating effect of gender is much more evident on the relationship between the video images of a tourist destination and online word of mouth.

**TABLE 7:** Moderating effect test for gender.

Hypothetical path	Path coefficient of population	Female	Male	Difference ratio	Results
H5: Perception of video images → online word of mouth	0.647***	0.713***	0.598***	17.7%	yes
H6: Online word of mouth → travel intentions	0.620***	0.629***	0.622***	1.1%	yes
H7: Perception of video images → travel intentions	0.130**	0.124	0.129	3.8%	no

**p < 0.01, ***p < 0.001.

## Discussion

The results of this study have shown that both the cognitive and affective video images of tourist destinations have a significant positive impact on the quality and quantity of online word of mouth (H1a, H1b, H1c, and H1d). This finding indicates that the better the cognitive and affective video images of a tourist destination, the higher the quality of online word of mouth contributed by tourists while sharing their travel experiences, and the greater the number of online word of mouth evaluations. This study verifies the assumption that the image of a tourist destination can lead to online word of mouth evaluations of it (Kim et al., 2017; Yang, 2018). Therefore, it is also concluded that the image of a tourist destination is one of the most important antecedent variables stimulating online word of mouth.

Among the hypotheses on the significant positive impacts of the perception of video images on tourists’ travel intentions, there is strong evidence for H2a; that is, the cognitive perception of video images has a notable positive impact on tourists’ travel intentions (de la Hoz-Correa and Muñoz-Leiva, 2019; Afshardoost and Eshaghi, 2020). The affective perception of video images does not have the same influence on tourists’ intentions to visit (H2b), probably because most tourists who upload travel videos to short-video platforms have few video shooting and editing skills. The intention to visit a destination cannot be effectively generated if viewers do not form a strong affective image perception of it while watching the short videos. This is similar to the findings of Tassiello and Tillotson (2020) and Ran et al. (2021). However, this study reaches the opposite conclusion that the affective image perception of a destination can impact tourists’ intentions to visit it, which has also been demonstrated in some of the existing literature (Han et al., 2019; Afshardoost and Eshaghi, 2020; Akgün et al., 2020).

Based on the analysis results of the hypotheses on the relationship between online word of mouth and tourists’ travel intentions, it is concluded that both the quantity and quality of online word of mouth have a positive impact on tourists’ intentions to visit (H3a and H3b). This may indicate that the higher the quality of online word of mouth reviews, the stronger the consumers’ sense of product quality, accelerating consumers’ decision-making behaviors. This is consistent with the findings of Nieto-García et al. (2017), Pourfakhimi et al. (2020), and Silaban et al. (2022), and also verifies that the quantity of online word of mouth evaluations can positively and directly affect tourists’ travel intentions, which is consistent with the conclusion of Wei (2020) and Xu and Yao (2020).

Hypotheses H4a and H4b on whether there is a mediating effect for online word of mouth on a destination’s cognitive image and tourists’ travel intentions have been verified. Hence, online word of mouth not only mediates the relationship between the cognitive and affective video images of tourist destinations and travel intentions, but can also convey the effects of the two to a significant extent (H4a and H4b). This research not only verifies that the emotional event of destination perception through video images can predict changes in tourists’ emotional evaluations and decision-making behaviors, but also proves that online word of mouth plays an important transmission role in the perception of cognitive and affective video images affecting tourists’ intentions to visit destinations. Other studies have come to similar conclusion, including Zhu and Hou (2013), Farrukh et al. (2020), and Junaedi and Harjanto (2020).

Regarding the moderating effect of gender, the following conclusion were reached. Gender differences have been detected between the influence paths of the perception of video images on online word of mouth (H5). Online word of mouth has a more significant impact on men and women when they watch short videos, forming cognitive and affective images of tourist destinations. Specifically, it was found that female potential tourists are more likely than males to pay attention to online word of mouth evaluations once they form a cognitive and affective perception of the image of a tourist destination generated by videos, which is similar to the findings of Wang et al. (2016). An analysis of the hypothesis on the moderating effect of gender between online word of mouth and tourists’ intentions to visit a destination (H6) also shows differences between men and women. These are mainly reflected in that female users attach more importance to online word of mouth evaluation, have a higher level of sensitivity to it, and are more likely to form travel intentions. This is similar to the findings of Asada and Ko (2019), which shows that female online platform users are more influenced by online word of mouth and are more likely to form the intention to act and make decisions. However, gender has no moderating effect on the relationship between the perception of video images and tourists’ travel intentions, contrary to the conclusion drawn by Huang and Veen (2019). That study suggests that gender does have a positive and significant effect on the image of a tourist destination and on tourists’ attitudes and behavioral intentions. However, the research results of Yang et al. (2020a) correspond to those of this study, indicating an interesting possibility that the moderating effect of gender may be significant if the sample size is expanded. Therefore, this study contends that if a certain sample size is employed, gender may play a significant moderating effect between the image of a tourist destination generated by videos and travel intentions. In summary, gender has a noticeable moderating effect between the image of a tourist destination generated by videos and online word of mouth.

## Conclusion

Through analyzing the behavior of users of short-video platforms, this study has comprehensively explored the dynamic of how the destination perception of video images influences tourists’ travel intentions. Using structural equation modeling, this study examines the behavior of 390 short video users who have watched travel videos. Based on the emotional evaluation theory, the mechanism of the mediating effect of the quality and quantity of online word of mouth on the relationship between the cognitive and affective perception of video images and tourists’ travel intentions is considered. The conclusion can be summarized as follows. First, the two dimensions of the perception of video images have a significant positive impact on the quality and quantity of online word of mouth, which in turn exerts a notable positive influence on tourists’ travel intentions. Among the two dimensions, only cognitive image perception positively affects travel intentions noticeably. Second, online word of mouth not only mediates the connection between the destination perception of video images and tourists’ travel intentions, but also significantly mediates the effects of the two. Third, gender has a clear and positive moderating effect on both the formation of the destination perception of video images and the online word of mouth of short-video platform users, with a larger effect on females than on males.

The theoretical contributions of this study are discussed as follows. First, this study contributes to the academic research in the field of video image perception by revealing how such image perception of destinations influences tourists’ intentions to visit them. While previous literature has examined the relationship between the two (Fang and Tang, 2020), it has failed to reveal the mutual or reciprocal influence on each other. Therefore, this study provides empirical evidence for the relationship between destination perception of video images and tourists’ travel intentions, using an investigation of short-video platform users, filling the research gap regarding short video images of tourist destinations.

Second, by incorporating online word of mouth as an intermediary variable into the research model, this study reveals relatively comprehensive relationships between the destination perception of video images and tourists’ travel intentions, thus enriching the research on online word of mouth and related fields. Furthermore, this study also emphasizes the moderating effect of gender. Based on the results, gender has a positive moderating effect (e.g., between the perception of video images and online word of mouth). Thus, this study forms the basis for more research on gender effect.

Additionally, the findings confirm that the emotional evaluation theory is appropriate for investigating the perception of video images, online word of mouth, and tourists’ travel intentions. Hence, this study can not only enrich the scope of application of emotional evaluation theory, but also contribute to the body of knowledge on destination marketing and management from a theoretical perspective.

The following suggestions and proposals are provided for tourism destination managers, enterprise operators, and other stakeholders. First, managers of tourism destinations and tourism-related enterprises should use short videos and social media platforms appropriately, and pay attention to the informative content presented by the videos. Hence, they can convey to potential tourists that their destinations have safe and standardized management procedures and comprehensive support service facilities. They should also convey distinctive local elements and the cultural connotations of their core attractions, attaching priority to the development of products and services that tourists can participate in and experience, thus establishing an attractive, cognitive image of their tourist destination. In addition, in the era of full-time media and self-media, most travel videos on short-video platforms are not uploaded by tourist officials or companies but are shot and shared by tourists themselves. However, most tourists are not professional videographers, thus their videos have deficiencies in terms of shooting angles and presentation techniques. Therefore, it is necessary for tourism destination managers and destination marketing agencies to highlight the affective connotations of the destination images presented in their videos. Doing so can convey the beautiful, pleasant, healthy, and positive aspects of the destination to potential consumers, which can instill in short-video platform users a strong intention to visit. It is also important to produce accurate and differentiated promotional videos covering the various dimensions of a destination. For example, aspects of a destination’s unique historical and folk culture can be included in promotional videos, as well as its distinctive natural environment. The special lifestyle and attractive food of local residents can also be presented to convey an atmosphere that is warm and hospitable to tourists, potentially forming an emotional connection between potential tourists and a destination, and then the strong intentions to visit might be generated in short-video platform users.

Second, the empirical research underpinning this study does not directly lead to the conclusion that the affective image of a tourist destination generated by the viewing of videos has a significant impact on tourists’ travel intentions. However, it can still indirectly influence them through the quality and quantity of the resulting online word of mouth, thereby strengthening tourists’ intentions to visit. Online word of mouth is a double-edged sword and should be monitored by relevant managers on a real-time basis. As long as the people concerned emphasize the quality and quantity of online word of mouth, ensure the inclusion of positive and reliable information, and strive to increase the exposure of this content, the persuasive influence of online word of mouth on tourists’ potential travel intentions can be maximized. Meanwhile, negative online word of mouth should be appropriately dealt with, and an analysis of the reasons for this negativity and possible improvements to address it can be performed through criticism and questioning. To deal with false information, tourism departments, enterprises, and management organizations can counter with accurate information and offer explanations through channels, including professional tourism websites and popular self-media and social media platforms. Thus, they can encourage these users to obtain information from official sources and to place trust in these sources and share their videos. Regarding incomplete information, supplementary explanations should be given promptly to establish a comprehensive and positive image of a given tourist destination through videos.

Last but not least, gender plays a significant moderating role in the dynamic involving travel videos, online word of mouth evaluations, and tourists’ travel intentions. This role mainly manifests in the gender differences exhibited in the construction of online word of mouth evaluations based on the video images of tourist destinations. It was shown that female users are more commonly affected by the perception of video images and are more focused on online word of mouth evaluations than males. Hence, the relevant departments of tourism management companies and other stakeholders (e.g., travel agencies, tourist attractions, hotels, etc.) should be cognizant of gender differences when formulating short video marketing plans. When seeking to attract female tourists, the priority should be to improve their perception of the image of the tourist destination that is generated through short videos. Although male consumers do not act like females, who are readily affected by online evaluations, it is still necessary to focus their attention on online word of mouth evaluations to avoid losing potential tourists.

This study has certain limitations. First, the research sample only included potential tourists who are drawn from a pool of short-video platform users rather than actual tourists; thus, it is imperative to extend further the scope of research in this field. Future research should conduct surveys of the diverse travel intentions of various groups and reduce bias by widening the scope of the sample and prolonging the time for respondents to answer. Second, based on emotional evaluation theory, this study discussed the dynamic through which the destination perception of video images affects tourists’ travel intentions. In this process, only the mediating effect of online word of mouth is analyzed, and not other possible factors. Therefore, other intermediary variables could be introduced to conduct comparative analyses in the future, enabling the development of more targeted market management strategies. Finally, short video content was not analyzed in this study. Future research may analyze tourists’ travel intentions according to video content types.

## Data availability statement

The original contributions presented in this study are included in the article/supplementary material, further inquiries can be directed to the corresponding author.

## Ethics statement

Ethical review and approval was not required for the study on human participants in accordance with the local legislation and institutional requirements. Written informed consent from the patients/participants or patients/participants legal guardian/next of kin was not required to participate in this study in accordance with the national legislation and the institutional requirements.

## Author contributions

YZ and LL contributed to conception and design of the study. YZ, LL, and XS wrote sections of the manuscript. All authors contributed to the article and approved the submitted version.
